# The effect of a proprietary chlorine-stabilizing surface-coating formulation on healthcare environment disinfection in multidrug-resistant bacteria isolation rooms

**DOI:** 10.1186/s13756-026-01782-z

**Published:** 2026-06-29

**Authors:** Yael Givon-Cohen, Nadav Abel, Tatiana Vander, Olga Libkind, Pasha Gur, Shahar Frechtman-Berger, Keren Anat Resnick, Jacob Moran-Gilad, Oren Zimhony

**Affiliations:** 1 Infectious Diseases Unit, Faculty of Medicine, Kaplan Medical Center, Hebrew University of Jerusalem, Pasternak St 1 Rehovot, Rehovot, POB1 76100 Israel; 2 Herzfeld Geriatric Rehabilitation Hospital, Gedera, Israel; 3 Faculty of Medicine, Hebrew University of Jerusalem, Jerusalem, Israel; 4 Department of Health Policy and Management, School of Public Health, Faculty of Health Sciences, Ben-Gurion University of the Negev, Beer-Sheva, Israel; 5 Clinical Microbiology Laboratory, Department of Clinical Microbiology and Infectious Diseases, Hadassah-Hebrew University Medical Center, Jerusalem, Israel

## Abstract

**Objective:**

OxiLast™ is a surface coating formulation that extends the antimicrobial effect of chlorine-based disinfectants. We sought to test it in a healthcare environment.

**Design:**

Controlled quasi-experimental study with parallel groups and repeated measures, conducted in isolation rooms of chronically ventilated patients, carriers of carbapenem-resistant *Acinetobacter baumannii* (CRAB).

**Methods:**

Standard sodium dichloroisocyanurate (NaDCC) disinfection was supplemented with OxiLast™ daily for 28 days at five patient stations, followed by twice-weekly application for 21 days. In parallel, six control stations were cleaned daily by NaDCC alone throughout the study. Environmental sampling targeted high touch surfaces adjacent to patient beds. Quantitative colony counts (CFU/cm2) and qualitative detection of CRAB and methicillin-resistant Staphylococcus aureus (MRSA) were performed at four time points: Day 0 (baseline), 21, 42, and 49. A decrease in environmental bioburden (target <5 CFU/cm^2^), CRAB and MRSA detection were defined as the primary outcomes. Achieving these benchmarks following twice-weekly application for three weeks was defined as the secondary outcome.

**Results:**

Baseline contamination averaged 1,272 CFU/cm^2^ (median 800 CFU/cm^2^) and 17,172 CFU/cm^2^ (median 756 CFU/cm^2^) in the control and intervention rooms, respectively. CRAB was detected in all stations of both groups, MRSA was detected in 4/6 and 5/5 of the control and intervention stations, respectively. On Day 21 following daily NaDCC-OxiLast™, all intervention stations dropped below 5 CFU/cm^2^ (mean 2.07 CFU/cm^2^). The control group averaged 316 CFU/cm^2^. CRAB and MRSA were detected in 5/6 of the control and in 1/5 and 0/5 in the intervention stations, respectively. Following twice-weekly NaDCC-OxiLast™ cleaning for 21 days, on Day 49, 4/5 intervention stations reached <5 CFU/cm^2^ (averaging 1.33 CFU/cm^2^), CRAB was undetected and MRSA was detected in 1/5 stations. All control stations remained >5 CFU/cm^2^, averaging 253 CFU/cm^2^, CRAB and MRSA were detected in 5/6 and 6/6 stations, respectively. At Days 21 and 49, the odds ratio for both CRAB and MRSA contamination in intervention rooms, was 0.026 (95% CI 0.00–0.43; *p* = 0.008).

**Conclusions:**

These findings provide proof-of-concept for an extended-action protocol using OxiLast™ adjunct to NaDCC. The impact of this intervention on MDRO transmission and clinical infections should be further studied.

**Supplementary Information:**

The online version contains supplementary material available at https://doi.org/10.1186/s13756-026-01782-z.

## Introduction

Healthcare-associated infections (HAIs) impose a substantial burden on hospitals, nursing homes, long-term care, and rehabilitation facilities. Patients become colonized with multidrug-resistant organisms (MDROs) for long periods [1–4]. Environmental contamination of high-touch surfaces contributes to indirect transmission via healthcare workers’ hands [1–4]. Enhanced environmental cleaning and disinfection interventions have been linked to reduced transmission of healthcare-associated pathogens and consequently reduced HAI incidence [5–7]. Nevertheless, persistent contamination occurs even after routine disinfection, due to rapid re-soiling, ongoing patient shedding, and limited residual activity of commonly used disinfectants [8–10]. 

Although no universally accepted microbiological standard exists, benchmarks based on aerobic colony counts (ACC) were suggested. Some recommend that high-touch surfaces should remain at or below 5 CFU/cm^2^ following effective cleaning [11]. Levels exceeding this benchmark signal inadequate hygiene and increased MDRO transmission risk [3]. 

Chlorine-based disinfectants, including sodium dichloroisocyanurate (NaDCC), remain a primary choice for healthcare surface disinfection due to broad-spectrum efficacy and rapid sporicidal activity (e.g., against *Clostridioides difficile*) [12, 13]. However, microbial loads rebound to pre-cleaning levels within hours, above the 5 CFU/cm^2^ threshold, due to a lack of sustained residual activity once dried [8, 10]. 

Logistic challenges further impede compliance with high-frequency cleaning. Environmental services staff often face workforce constraints, competing demands, and variability in adherence to procedure standards [12, 14]. Maintaining surfaces below 5 CFU/cm^2^ between routine cleaning cycles is especially difficult in units caring for patients colonized with methicillin-resistant *Staphylococcus aureus* (MRSA), Vancomycin-resistant enterococci (VRE), carbapenem-resistant *Acinetobacter baumannii* (CRAB), or *Candidozyma auris.* [8, 9, 12] These challenges highlight the need for adjunctive strategies that enhance and prolong bactericidal activity on disinfected surfaces without adding workload.

Extended-action disinfectants based on quaternary ammonium (QA) have been proposed to address this gap by leaving a thin layer that slowly releases the active agent [15, 16]. OxiLast™ is a liquid companion formulation for on-site mixing with NaDCC, extending chlorine surface activity from hours to days without changing workflows [17]. Maran et al. demonstrated that OxiLast™ coating of surfaces in unoccupied patient rooms confers sustained reduction in bacterial counts of inoculated MDROs following routine NaDCC disinfection, up to 7 days following a single application of OxiLast™ [18].

We further evaluated the microbiological impact of adding OxiLast™ to routine NaDCC-based disinfection in occupied isolation rooms at a long-term care facility (LTCF), assessing the reduction in bioburden to less than 5 CFU/cm^2^ and that of MDROs to below detection level. We also tested whether this effect is sustained following a transition from daily to twice-weekly application.

## Methods

### Study design and setting

A single-center, controlled, quasi-experimental study with parallel groups and repeated measures was conducted during 2025 in CRAB cohorting isolation rooms of chronically ventilated patients in a long-term care facility in Israel. At baseline, the patient environment was disinfected with NaDCC (2000 ppm) according to facility practice, with routine disinfection performed on weekdays and not on weekends (Friday-Saturday). Sampled patient stations were occupied by the same patient throughout the study period, from baseline to Day 49, to minimize inter-patient variability in shedding. Stations were anonymized and designated as Units A, B1, B2, C, and so forth.

### Intervention protocol

The intervention timeline, detailed disinfection protocols, and sampling regimen for both groups are summarized in Table 1. Control surfaces were disinfected with NaDCC throughout the study according to standard facility practice. The intervention group followed a two-stage protocol: 28 days of daily NaDCC-OxiLast™ application, followed by 21 days of twice-weekly NaDCC-OxiLast™ application.

**Table 1:** Study timeline and disinfection protocol in intervention and control groups, including cleaning frequency and environmental sampling time points

Study day	Stage	Control group protocol (*n* = 6 stations)	Intervention group protocol (*n* = 5 stations)	Environmental sampling
Day 0	Baseline	Daily NaDCC (2000 ppm)	Daily NaDCC (2000 ppm)	Baseline Sampling
Days 1–28	*Phase 1: Daily* Application	Daily NaDCC (2000 ppm)	*Daily* NaDCC-OxiLast™ (2000 ppm)	Routine Disinfection
Day 21	Post-Intervention (Week 3)	Daily NaDCC (2000 ppm)	Daily NaDCC-OxiLast™ (2000 ppm)	1st sampling
Day 28	Protocol Change	Daily NaDCC (2000 ppm)	Frequency reduced to: Twice-Weekly NaDCC-OxiLast™	No Sampling
Day 42	Reduced Frequency (two weeks)	Daily NaDCC (2000 ppm)	Twice -Weekly NaDCC-OxiLast™	2nd Sampling
Day 49	Reduced Frequency (three weeks)	Daily NaDCC (2000 ppm)	Twice Weekly NaDCC-OxiLast™	3rd, final Sampling

During the second phase, no scheduled cleaning or disinfection was performed between the twice-weekly NaDCC-OxiLast™ applications. During both phases, visible soiling, body-fluid contamination, or immediate care-related contamination was cleaned as needed according to standard facility practice.

No formal cleaning-time measurements or cleaning-compliance audits were performed to compare cleaning thoroughness between groups. Application of the NaDCC-OxiLast™ formulation was not blinded to the cleaning staff or to the sampling operator. Cleaning staff in both groups were aware that disinfection efficacy was being monitored, but were not informed of the expected results. Laboratory personnel was not informed regarding the study groups.

The study was reviewed by the institutional Ethics committee, which determined that formal IRB approval was not required.

### Environmental sampling and laboratory analysis

Environmental sampling was conducted on Sundays between 07:00 and 09:00, before routine daily disinfection, to capture residual contamination after the weekend interval. Sampling targeted predefined high-touch, non-porous surfaces at each patient station, including inner and outer bed rails nearest to the patient, control panels, handles, and call buttons. To ensure reproducibility, sampling was limited to mapped areas and was performed throughout the study by the same trained operator who knew the intervention.

At each patient station, a standardized total surface area of 900 cm² was sampled. The sampling area was measured using rulers to ensure consistent surface size across sampling events. Samples were collected using individually packaged sterile StickSponge™ polyurethane sponges pre-moistened with 10 mL neutralizing buffer (containing sodium thiosulfate) (Hygiena^®^ HSB100NBP). The sponge was applied over the defined area using a multidirectional technique to ensure complete coverage, with consistent pressure applied while preserving the soaking buffer. Samples were immediately refrigerated and transported to the laboratory under controlled temperature. Upon receipt, each sponge was manually compressed within its original bag to recover the maximum available fluid. The recovered fluid was transferred into a sterile 15 mL conical tube for individual processing.

For quantitative bacterial load analysis, serial ten-fold dilutions of the recovered fluid were performed up to a factor of 10⁶. A volume of 100 µL from each dilution was plated onto Tryptic Soy Agar (TSA) and incubated overnight at 37 °C. CFUs were enumerated from plates within the countable range of 20–200 CFU. CFU calculations were based on the original sponge fluid volume of 10 mL, and total bacterial load was expressed as CFU/cm² by dividing the calculated CFU count by the sampled area of 900 cm². Under these conditions, a single colony represented 100 CFU per sampled area, corresponding to 10 CFU/mL and 0.11 CFU/cm². Samples without detectable bacterial growth were reported as below the limit of detection.

For qualitative MDRO detection, undiluted recovered fluid was plated onto selective media for CRAB detection using CHROMagar mSuperCARBA (HyLabs, Rehovot, Israel) and for MRSA detection using CHROMagar MRSA (HyLabs, Rehovot, Israel). Selective culture was performed using direct plating as well as plating after overnight enrichment in tryptic soy broth. Suspected isolates were identified morphologically according to the manufacturers’ instructions and confirmed using MALDI-TOF mass spectrometry (VITEK MS, bioMérieux, Lyon, France).

### Statistical analysis

The primary outcomes were reduction of bacterial bioburden to below the predefined threshold of 5 CFU/cm² and reduced detection of CRAB and MRSA from sampled surfaces. Sustained reduction in bacterial bioburden following transition to twice-weekly NaDCC-OxiLast™ application while the control group continued standard NaDCC disinfection was defined as a secondary exploratory outcome.

As this was a controlled, quasi-experimental study with parallel groups and repeated measures, the number of sampled stations was determined by the available CRAB-cohorting isolation rooms meeting eligibility criteria rather than by an a priori power calculation. Stations were assigned without randomization or stratification by patient characteristics. With five intervention stations and six control stations, and a pooled standard deviation of 0.67 log_10_ CFU/cm² estimated from the observed data, a two-sided two-sample comparison at α = 0.05 provided post hoc 80% power to detect a minimum between-group difference of 1.13 log₁₀ CFU/cm² and 90% power to detect a difference of 1.31 log_10_ CFU/cm².

Bacterial burden was expressed as CFU/cm². Values with no detectable bacterial growth were reported as ‘below the limit of detection’. For statistical analysis, these values were assigned a value of zero. Because log₁₀(0) is undefined, a pseudo-count of 1 CFU/cm² was added before transformation. The outcome variable was therefore calculated as log₁₀(CFU/cm² + 1).

Differences in log₁₀(CFU/cm² + 1) are reported between-group comparisons on the same sampling days and within-group between sequential samplings. Group differences over time were evaluated using linear mixed-effects models with fixed effects for group, time, and their interaction, and a random intercept for sampled station to account for repeated measurements within stations. Time was treated as a categorical variable with four levels: Day 0, Day 21, Day 42, and Day 49. Type III analysis of variance was used to assess the significance of fixed effects. Post hoc pairwise comparisons between groups at each time point and within groups across time were derived from the mixed model, with Holm-adjusted *p*-values to control for multiple testing. Model assumptions were evaluated using residual diagnostics.

To evaluate robustness and address baseline imbalance, a sensitivity analysis was performed, excluding a sampled station with extreme baseline contamination.

Between-group differences in MDRO detection at each time point were assessed using Fisher’s exact test. Odds ratios comparing intervention and control stations were estimated using Firth penalized logistic regression because of the small sample size and complete separation in some comparisons. All tests were two-sided, and *p*-values < 0.05 were considered statistically significant. Analyses were performed using R (R Foundation for Statistical Computing, Vienna, Austria).

## Results

The study included the same five and six patient environment stations for the intervention and control group, respectively. The results of each sampling station at each sampling time point, are presented in Table S1.

Baseline contamination averaged 1272 CFU/cm^2^ (ranging 56–4333, median 800 CFU/cm^2^) and 17,172 CFU/cm^2^ (ranging 286–83,333, median 756 CFU/cm^2^) in the control and intervention stations, respectively. An outlier CFU/cm^2^ value in intervention station H2 (Table S1) accounted for the higher mean of that station. CRAB was detected in all stations of both groups.

Between-group analysis comparing intervention with control at each time point using Holm-adjusted pairwise contrasts showed no statistically significant difference at baseline log₁₀(CFU/cm² + 1) 0.455 (95% CI: − 0.295 to 1.204, *p* = 0.223).

### Primary outcome daily application for control and intervention group

**Fig. 1:**
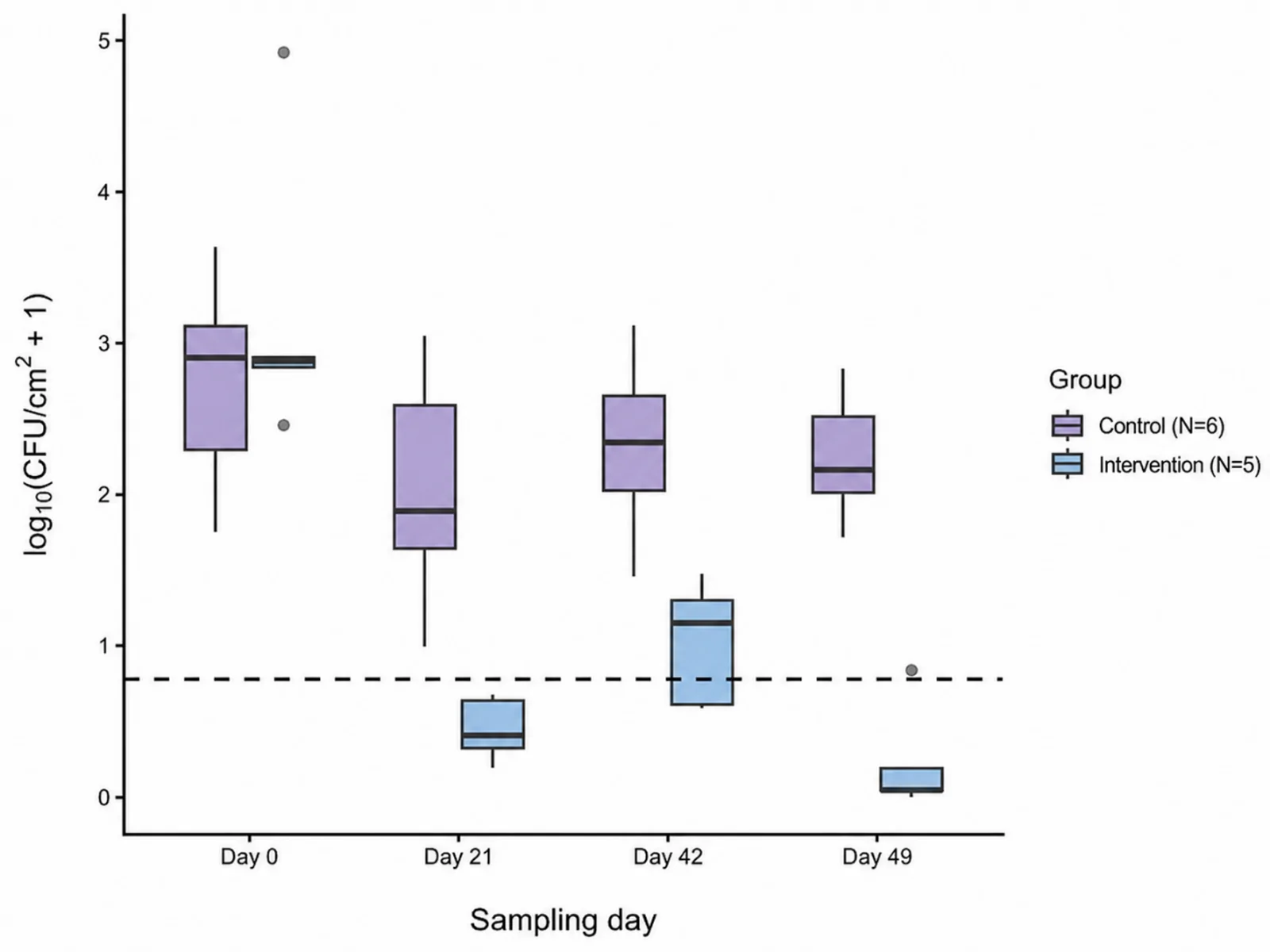
Bacterial Load Distributions Across Time Points in intervention and control stations. Boxplots of log_10_ CFU/cm²+1 on high-touch surfaces in five intervention and six control rooms at days 0, 21, 42, and 49. Boxes indicate the interquartile range with median values; whiskers represent 1.5 × IQR. Dashed line displays log_10_ log_10_ (5 + 1) CFU/cm^2^

For Intervention group, at Day 21, all sampled surfaces dropped below 5 CFU/cm² (mean 2.07 CFU/cm²; log₁₀ (CFU/cm²+1), reduction range 2.14–4.51, mean Log_10_ (CFU/cm²+1) for a reduction of 2.73 (Fig. 1), whereas none of the stations in the control group dropped below 5 CFU/cm², averaging 316 CFU/cm² ,mean log₁₀(CFU/cm² + 1), for a reduction of 0.73.

Following 21 days, the intervention resulted in a significant reduction in bioburden log₁₀(CFU/cm² + 1) 1.585 (95% CI:0.69 to 2.5, *p* = 0.002) compared to control. To exclude regression towards the mean for an outlier measurement of H2 station as an explanation for the difference, we conducted a sensitivity analysis following exclusion of H2 results, resulting in a drop of log_10_ (CFU/cm^2^+1)1.57 (95%CI 0.87 to 2.28- *p* < 0.001).

Within-group analysis of the control group using pairwise comparisons between all time points showed no significant changes in bacterial load over time (p-values > 0.12). For the intervention group, bacterial load decreased significantly from Day 0 to Day 21 log₁₀(CFU/cm² + 1), 2.752 (95% CI: 1.876 to 3.628, *p* < 0.001).

MDRO detection (CRAB and MRSA) (Table S1) was similar at baseline: CRAB 6/6 and 5/5, and MRSA 4/6 and 5/5, for the control and intervention groups, respectively. On Day 21 it was 5/6 for both CRAB and MRSA in the control group, and 1/5 CRAB and 0/5 MRSA for the intervention group *p* = 0.047, OR = 0.14 (95%CI: 0.02–0.97).

### Secondary outcome (Daily NaDCC vs. Twice-weekly intervention)

Following a shift in cleaning frequency to twice-weekly, on Day 42 sampling, three stations in the intervention group transiently rebounded above 5 CFU/cm^2^ (group average of 13.4, range 2.9–28.9 CFU/cm^2^), while contamination levels in the control group stations remained above the threshold (average 401, range 28−1300 CFU/cm^2^). On Day 49, 4/5 intervention stations reached < 5 CFU/cm^2^ (average 1.33 CFU/cm^2^, range 0.46–2.12 CFU/cm^2^), compared to none of the control room stations (average 253 CFU/cm^2^ range 51–678 CFU/cm^2^).

Between-group comparison at Day 42, following a change to the twice-weekly course, revealed a significant decrease in log₁₀(CFU/cm² + 1) 1.297( 95% CI 0.547: to 2.046, *p* = 0.001) compared to control. The largest reduction was observed following another week at Day 49, log₁₀(CFU/cm² + 1). 2.019 (95% CI: 1.270 to 2.769, *p* < 0.001).

Within-group analysis of the intervention group showed a non-significant increase in bacterial load between Day 21 and Day 42, log₁₀(CFU/cm² + 1) − 0.578( 95% CI: −1.453 to 0.298, *p* = 0.143), indicating partial rebound following the initial reduction. This was followed by a further decline between Day 42 and Day 49 resulting in the lowest bacterial load at the end of follow-up on day 49 compared to baseline, log₁₀(CFU/cm² + 1) 2.976 (95% CI:2.100 to 3.851, *p* < 0.001).

No significant difference was evident between Day 21 and Day 49 for the intervention group log₁₀(CFU/cm² + 1) 0.224( 95% CI − 0.652-to 1.1 *p* = 0.473).

Following two weeks of twice-weekly cleaning, at Day 42, both CRAB and MRSA were detected in all stations in the control group (6/6), and in 3/5 of the intervention group (*p* = 0.52), indicating a rebounded detection of MDRO in correlation with the transient bioburden increase. Nevertheless, following another week at Day 49, CRAB and MRSA were detected in 5/6 and 6/6, and 0/5 and 1/5 of the control and intervention stations, respectively, *p* = 0.023, OR = 0.13, (95% CI 0.019–0.88).

Firth penalized logistic regression for CRAB and MRSA detection yielded odds ratios (OR) 0.026 for intervention versus control at either Days 21 or 49 ; *p* = 0.008 (95% CI 0 to 0.434), indicating a marked reduction in the probability of significant contamination at these follow-up points.

**Fig. 2:**
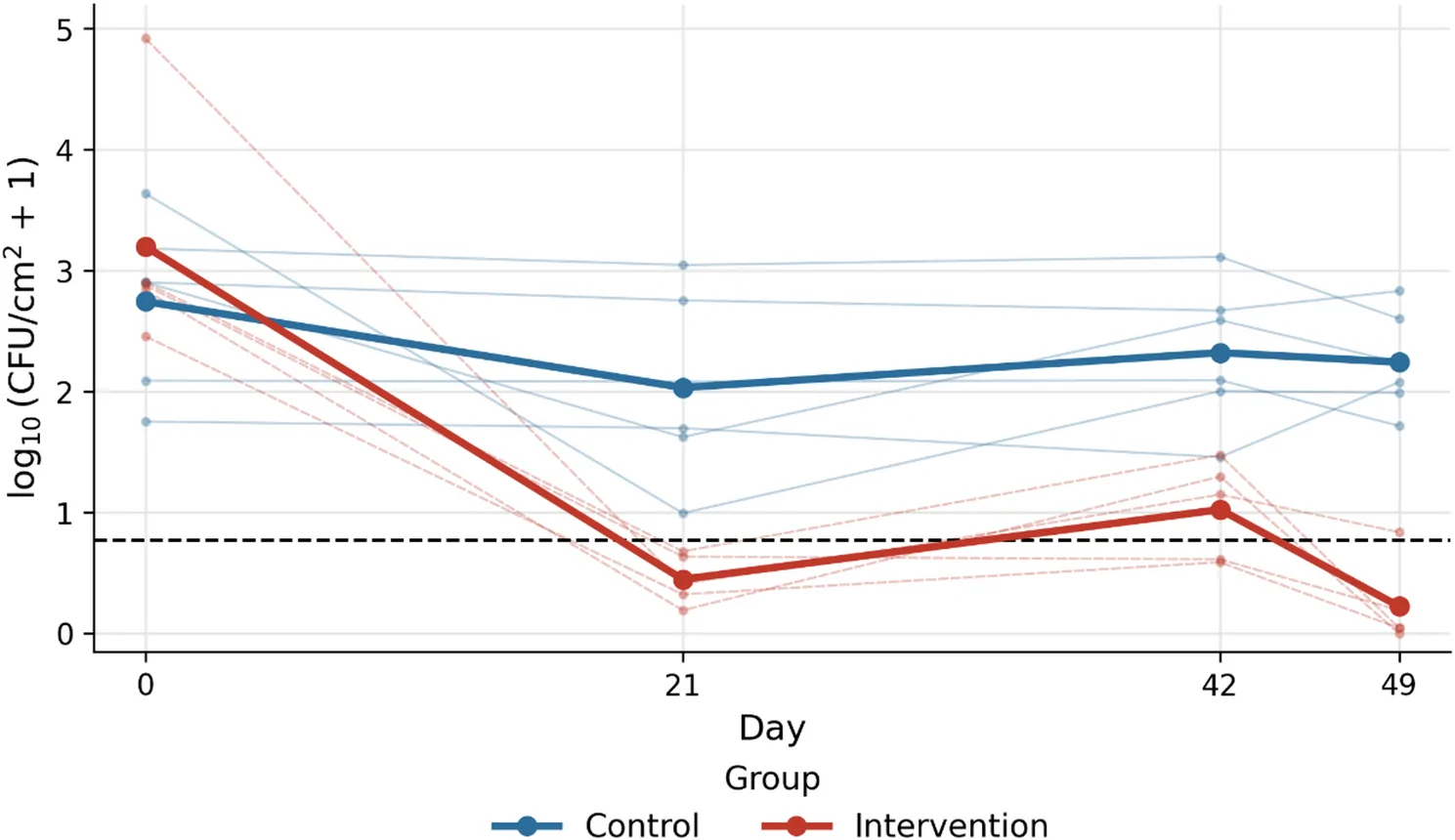
Differential temporal patterns of bacterial load in intervention versus control stations. Temporal trajectories of log_10_ CFU/cm²+1 on high-touch surfaces in intervention and control stations at days 0, 21, 42, and 49. Dashed line displays log_10_ (5 + 1) CFU/cm^2^. Thin lines represent individual environmental stations; bold lines represent group means

A linear mixed-effects model with fixed effects for group, time, and their interaction and a random intercept for surface was fitted to log₁₀-transformed bacterial counts calculated as log₁₀(CFU/cm² + 1). Type III analysis of variance demonstrated a statistically significant main effect of group (F = 18.165, *p* = 0.002), indicating an overall difference in bacterial load between the intervention and control groups. A strong effect of time was also observed (F = 31.076, *p* < 0.001), reflecting substantial temporal changes in bacterial load. Importantly, a highly significant group × time interaction was detected (F = 13.587, *p* < 0.001), indicating that the trajectory of bacterial load over time differed markedly between groups. From Day 21 and on days 42 and 49, bacterial load was consistently and significantly lower in the intervention group, with the largest difference at Day 49, corresponding to a more than 100-fold reduction compared with control (Fig. 2).

## Discussion

NaDCC-OxiLast™ extended-action protocol was associated with a reduction in environmental bioburden in intervention rooms, with most sampled surfaces meeting the predefined < 5 CFU/cm² benchmark, accompanied by a corresponding decrease in CRAB and MRSA detection. In contrast, standard daily NaDCC disinfection in the control group was not associated with sustained reduction below this cutoff, underscoring the limitations of conventional, short-acting chlorine-based disinfectants in high-burden environments [1–4]. In the control group, the Day 0 to Day 21 change was modest and not statistically significant (Δ = 0.712 log₁₀[CFU/cm² + 1], *p* = 0.104), and may reflect background variability or a Hawthorne effect related to monitoring. The reduction observed at intervention stations during the first phase, when cleaning frequency was identical in both groups, is consistent with a prolonged chlorine activity on treated surfaces through the companion formulation intended to sustain NaDCC activity. Notably, this intervention was implemented within the existing environmental disinfection workflow [17]. 

Moreover, the results in the control group highlight the well-recognized gap between protocol and real-world efficacy [11, 14]. Environmental contamination in long-term care and rehabilitation facilities is driven by intensive care needs, shared equipment, and prolonged MDRO carriage [1–4]. The immobility of chronically ventilated patients necessitates bed baths and diaper changes within the patient unit, which may contribute to shedding of skin microbiota and pathogens onto surrounding surfaces [2–4]. 

Continuously active disinfectants and antimicrobial surface coatings have been described previously for quaternary-ammonium and have been evaluated primarily in laboratory models and on portable medical equipment. A study on portable equipment reported significant reductions in aerobic colony counts over 7 days and reduced recovery of *Staphylococcus aureus* and enterococci [15]. A study comparing disinfection by quaternary-ammonium with and without extended activity in two ambulatory locations found no difference in bioburden on high-touch surfaces over 24 h [16]. Chlorine-based disinfectants are short-acting agents that lose activity shortly after drying, allowing microbial loads to rebound rapidly, often returning to pre-cleaning levels within hours despite guideline-concordant application [8, 10, 12]. 

Available evidence on chlorine surface disinfection is based almost entirely on short contact times of minutes to hours, with no established clinical evidence of sustained residual activity on dry surfaces under routine conditions [13]. So far, there are no field studies of surface-coating formulations specifically designed to prevent rapid loss of chlorine activity and prolong its bactericidal effect on high-touch healthcare surfaces. The formulation evaluated herein first demonstrated sustained antimicrobial activity in laboratory models and in simulated bacterial contamination of an unoccupied hospital room [18]. To our knowledge, the present study is the first real-life evaluation of a formulation intended to impede chlorine evaporation and extend residual activity in occupied patient rooms, isolation rooms caring for chronically ventilated CRAB carriers. The study was not designed to assess contact-time killing kinetics. The findings should therefore be interpreted as reduced rebound or re-accumulation of surface bioburden under routine conditions rather than as a direct comparison of immediate bactericidal killing rates.

The clearest assessment of the independent effect of adding OxiLast™ to routine NaDCC disinfection was observed during the first phase, when cleaning frequency was identical in both groups. By Day 21, bacterial load was significantly lower in the intervention group compared to the control and in comparison with the baseline in the intervention stations.

During the second phase, the intervention group transitioned to twice-weekly NaDCC-OxiLast™ application with no scheduled disinfection in between, while the control group continued standard daily NaDCC disinfection. Because cleaning frequency differed between groups during this phase, these later findings reflect the combined effect of the formulation and reduced scheduled disinfection and should be interpreted as secondary and exploratory rather than as evidence of the formulation’s independent effect. Nevertheless, bacterial load remained significantly lower in the intervention group than in the control group at Day 42 and Day 49, corresponding to approximately 20-fold and 105-fold reductions, respectively. This trajectory is consistent with the possibility that the extended-action protocol may help maintain low bioburden under reduced application frequency, a hypothesis that warrants future studies.

Notably, the reduction in total bioburden was paralleled by a marked decrease in MDRO prevalence on high-touch surfaces, in accord with other studies [8, 11, 14]. These findings support an association between reduced total bioburden and reduced MDRO recovery. However an effect on MDRO transmission, patient acquisition, infection, or healthcare-associated infection rates has yet to be examined.

The extended-action approach evaluated here focuses on prolonging the activity of the existing chlorine-based disinfectant itself rather than enhanced cleaning programs that rely primarily on increased frequency, audit-and-feedback, or novel application methods. Therefore, this approach may be easier to integrate into routine practice without major changes in staff behavior and possibly easier to sustain over time [15, 16]. Thus, this strategy may offer a practical way to maintain or improve environmental hygiene while reducing application frequency and dependence on perfect procedural adherence [8, 9, 12, 14].

This study has several limitations. First, it was conducted in a single long-term care facility among chronically ventilated CRAB carriers. Thus, the findings may not be generalizable to acute-care hospitals, ambulatory settings, non-ventilated patients, or other MDRO populations. Second, the study included a small number of environmental stations and was not randomized, stratified, blinded, or designed as a crossover trial. Patient placement and room allocation were determined by routine cohorting and infection-control practice, limiting causal inference. Additionally, cleaning staff and the sampling operator were not knew the intervention. It should be noted that decreased chlorine odor was reported by the intervention stations’ staff, a factor which may preclude the blinded application of this agent in further studies. Third, no formal audit of cleaning duration, cleaning thoroughness, or cleaning compliance was performed; therefore, performance or observation bias cannot be fully excluded. Fourth, baseline contamination was imbalanced because of one station with extreme baseline bioburden. However, Sensitivity analysis with exclusion of this station did not essentially change the results and conclusions and, argues against regression to the mean as a sufficient explanation for the observed effect. Finally, the study measured environmental bioburden and MDRO recovery from high-touch surfaces, not patient acquisition, transmission events, infection, or healthcare-associated infection outcomes. Longer follow-up, additional repeated sampling points, and randomized studies are needed to confirm durability, generalizability, and potential clinical relevance.

## Conclusion

Long-acting chlorine activity on healthcare surfaces may represent a promising adjunct to standard disinfection protocols, with the potential to enhance environmental hygiene in high-risk care settings while potentially allowing reduced application frequency and easing operational burden. These findings should be interpreted as hypothesis-generating and as providing proof-of-concept environmental microbiological evidence from a single high-burden long-term care setting. The effect of this modality on MDRO transmission, patient acquisition, and healthcare-associated infections remains to be prospectively evaluated.

## Electronic Supplementary Material

Below is the link to the electronic supplementary material.

Supplementary information

## Data Availability

All data generated or analyzed during this study are included in this published article and its supplementary information files (Supplement Table 1).

## Declarations

### Use of AI

Scientific Literature searches were supported by Perplexity AI, and scientific language editing was assisted by Gemini (Google) and Claude (Anthropic); the final version was proofread and approved by the authors.
