# Supplementary material for: The effect of a proprietary chlorine-stabilizing surface-coating formulation on healthcare environment disinfection in multidrug-resistant bacteria isolation rooms
Source: Antimicrob Resist Infect Control. 2026 Jun 29;15:117. doi: 10.1186/s13756-026-01782-z (PMC13613773; doi:10.1186/s13756-026-01782-z)
Supplement: Supplementary file 1 [file 13756_2026_1782_MOESM1_ESM.docx]

| **Group** |  | **0 baseline** | | | | **Day 21** | | | | **Day 42** | | | | **Day 49** | | | |
| --- | --- | --- | --- | --- | --- | --- | --- | --- | --- | --- | --- | --- | --- | --- | --- | --- | --- |
| **Control** | **Station** | **MRSA** | **CRAB** | **CFU/ml** | **CFU/cm^2^** | **MRSA** | **CRAB** | **CFU/ml** | **CFU/cm^2^** | **MRSA** | **CRAB** | **CFU/mL** | **CFU/cm^2^** | **MRSA** | **CRAB** | **CFU/mL** | **CFU/cm^2^** |
|  | **A1** | V | V | 11000 | 122.22 | V | X | 10800 | 120.00 | V | V | 11100 | 123.33 | V | X | 4600 | 51.11 |
|  | **B1** | V | V | 5000 | 55.56 | V | V | 4400 | 48.89 | V | V | 2500 | 27.78 | V | V | 10700 | 118.89 |
|  | **C1** | V | V | 390000 | 4333.33 | X | V | 800 | 8.89 | V | V | 9000 | 100.00 | V | V | 8700 | 96.67 |
|  | **C2** | X | V | 72000 | 800.00 | V | V | 3700 | 41.11 | V | V | 35000 | 388.89 | V | V | 15600 | 173.33 |
|  | **D1** | X | V | 72000 | 800.00 | V | V | 51000 | 566.67 | V | V | 42000 | 466.67 | V | V | 61000 | 677.78 |
|  | **D2** | V | V | 137000 | 1522.22 | V | V | 100000 | 1111.11 | V | V | 117000 | 1300.00 | V | V | 36000 | 400.00 |
| **Oxilast** | **E1** | V | V | 25700 | 285.56 | X | X | 100 | 1.11 | V | V | 260 | 2.89 | X | X | 10 | 0.11 |
|  | **F1** | V | V | 62000 | 688.89 | X | X | 50 | 0.56 | V | V | 1700 | 18.89 | X | X | *<LOD | *<LOD |
|  | **G1** | V | V | 68000 | 755.56 | X | V | 300 | 3.33 | X | X | 280 | 3.11 | X | X | 50 | 0.56 |
|  | **H1** | V | V | 72000 | 800.00 | X | X | 340 | 3.78 | V | V | 2600 | 28.89 | V | X | 10 | 0.11 |
|  | **H2** | V | V | 7500000 | 83333.33 | X | X | 140 | 1.56 | X | X | 1180 | 13.11 | X | X | 530 | 5.89 |

**Supplemental Table 1.** Raw bacterial count data

Raw data of CFU/mL, CFU/cm^2^,CRAB and MRSA detection in control and OxiLast™ stations in four sampling time points of the study. V- detection, X-no detection of MRSA or CRAB.

*LOD< implies less than 10 CFU/mL and 0.11 CFU/cm².
